# Impact of osteoporosis and vertebral fractures on quality-of-life. a population-based study in Valencia, Spain (The FRAVO Study)

**DOI:** 10.1186/1477-7525-9-20

**Published:** 2011-04-06

**Authors:** José Sanfélix-Genovés, Isabel Hurtado, Gabriel Sanfélix-Gimeno, Begoña Reig-Molla, Salvador Peiró

**Affiliations:** 1 Centro Superior de Investigación en Salud Pública (CSISP), Valencia, Spain; 2 Centro de Salud de Nazaret, Agencia Valenciana de la Salud. Valencia, Spain; 3 Centro de Salud de Villamarchante, Agencia Valenciana de la Salud. Valencia, Spain

## Abstract

**Background:**

To describe the health related quality of life in a population sample of postmenopausal women over the age of 50 and resident in the city of Valencia (Spain), according to the presence/absence of osteoporosis and the severity of prevalent morphometric vertebral fractures.

**Methods:**

A cross-sectional age-stratified population-based sample of 804 postmenopausal women of 50 years of age and older were assessed with the SF-12 questionnaire. Information about demographic features, lifestyle, clinical features, educational level, anti-osteoporotic and other treatments, comorbidities and risk factors for osteoporosis were collected using an interviewer-administered questionnaire and densitometric evaluation of spine and hip and spine x-rays were carried out.

**Results:**

In the non-adjusted analysis, mild and moderate-severe vertebral fractures were associated with decreased scores in the SF-12 Physical Component Summary (PCS) but not in the Mental Component Summary (MCS), while densitometric osteoporosis with no accompanying fracture was not associated with a worse health related quality of life. In multivariate analysis worse PCS scores were associated to the age groups over 70 (-2.43 for 70-74 group and -2.97 for 75 and older), chronic conditions (-4.66, -6.79 and -11.8 according to the presence of 1, 2 or at least 3 conditions), obesity (-5.35), peripheral fracture antecedents (-3.28), hypoestrogenism antecedents (-2.61) and the presence of vertebral fracture (-2.05).

**Conclusions:**

After adjusting for confounding factors, the physical components of health related quality of life were significantly lower in women with prevalent osteoporotic vertebral fractures than in women -osteoporotic or not- without vertebral fractures.

## Introduction

Osteoporosis is a common condition characterized by decreased bone mass and increased susceptibility to fractures [1]. The most common clinical complications of osteoporosis are hip, wrist, and vertebral fractures. Vertebral fractures (VFX) are the most prevalent osteoporosis-related fractures but they are often asymptomatic, and their underdiagnosis and undertreatment is well documented [2,3].

Measures of Health Related Quality of Life (HRQoL) have gained increasing attention as relevant outcomes in clinical studies of osteoporosis [4,5]. These measures are also used in epidemiological surveys, complementary to data on morbidity and health care utilization, to estimate the burden of disease and often to compare with other chronic diseases. Several instruments, both generic and disease targeted, have been used to examine HRQoL in osteoporosis and osteoporotic fractures [5-7]. The specific instruments most widely used include the Osteoporosis Quality of Life Questionnaire (OQLQ) [6,7] and its reduced version the mini-OQLQ [8], the Quality of Life Questionnaire of the European Foundation for Osteoporosis (QUALEFFO) [9,10], the Osteoporosis Assessment Questionnaire (OPAQ) [11,12], the Osteoporosis-Targeted Quality of Life Questionnaire (OPTQoL) [13,14] and the assessment of health-related quality of life in osteoporosis (ECOS-16) [15]. Among the generic instruments, those most used in osteoporotic patients includes the EuroQol 5-D (EQ5D) [16,17], the Medical Outcomes Study Survey Form (MOS-SF) in its SF-12 [18] or SF-36 [16] versions that could be combined with the disease-specific module Quality of Life in Osteoporosis (QUALIOST)[19,20], and the Health Utility Index [7,21].

Vertebral fractures and deformities result in back pain, disability, limitations in physical functioning and psychosocial impairment [22]. An increasing amount of literature has shown the relation between prevalent VFX (their number, severity and, occasionally, lumbar localization) and HRQoL decline [5,18,23-26]. Lower HRQoL has also been associated with incident VFX, with or without clinical manifestations [5,27-29]. However, the association with osteoporosis in the absence of fracture or with only mild morphometric fractures has been less studied. The aim of this study is to describe the HRQoL in a population sample of postmenopausal women of 50 years old and over and resident in the city of Valencia (Spain), according the presence/absence of osteoporosis and the severity of prevalent morphometric vertebral fractures.

## Methods

### Design

Population-based cross-sectional study conducted between February 2006 and March 2007, designed primarily to estimate the prevalence of densitometric osteoporosis and vertebral fracture.

### Population and simple

The study's population was post-menopausal women over the age of 50 living in the city of Valencia, Spain, excluding women with cognitive impairment, physical impediments preventing women from going to the radiology centre by her own means, race other than Caucasian and unwillingness to participate in the study. The methods of the FRAVO study, mainly designed to estimate the population prevalence of vertebral fracture and densitometric osteoporosis, have been fully described elsewhere [30]. Briefly, 1,758 women were selected from a simple age-stratified (50-54, 55-59, 60-64, 65-69, 70-74 y 75+) random sample from among the residents of Valencia, and invited to participate in the study. Only 1,314 confirmed receipt of the letter (74.7%) and of these, 76 presented at least one exclusion criteria, 371 declined to participate and 43 did not keep their appointments for the examinations, leaving 824 women participating in the study. In 19 cases the spine x-ray or the densitometry was not available and in 1 case the HRQoL questionnaire was not entirely fulfilled, leaving 804 women for analysis (dropouts by reason and age groups are described in Additional file 1).

### Main outcome measure

Health related quality of life was measured with the Spanish version-2 of the MOS SF-12 questionnaire [31], a simplified self-administered version of the SF-36 that could be completed within two minutes. The SF-12 is a generic instrument consisting of 12 items covering the domains of physical functioning, role limitations due to physical health problems, bodily pain, general health, vitality, social functioning, role limitations due to emotional problems and mental health. These domains can be summarized into a physical component summary scale (PCS-12) and a mental component summary scale (MCS-12). In the SF-12 version-2 for each one of the 8 domains and the summary components, items are aggregated and transformed into a 0 to 100 score, a low score indicating a lower HRQoL. To facilitate interpretation, the PCS and MCS scores are standardized with population norms, 50 (SD: 10) being the average of the general population [31]. Because Spanish weights were not available for the SF-12v2 at the time of analysis, we use the North American weights. Figures higher or lower than 50 should be interpreted as better or worse HRQoL scores than the reference population.

### Other variables and definitions

Information about demographic features, lifestyle, clinical features, educational level, anti-osteoporotic and other treatments, comorbidities and risk factors for osteoporosis was collected using an interviewer-administered questionnaire. Among other variables, it included the subject's age, place of birth, educational level (no studies, primary, secondary/university, and unknown studies), obesity grade II or more (body mass index (BMI)>35), hypoestrogenism antecedents (menopause before age 40 and/or amenorrhea for more than a year) and asked whether the subject had a history of osteoporotic fracture excluding major traumatisms in any location. Using the information on risk factors, comorbidities and treatments, we constructed a variable to account for the presence of chronic conditions that could affect the HRQoL: taking corticoids for at least 3 months in the last year, gait abnormalities for any reason (or postural instability, impaired balance or anticonvulsive treatment), cognitive or visual deficit, depression (or taking lithium), and specific self-referred conditions such as gastrectomy, bowel resection, inflammatory bowel disease, thyroidectomy (or taking thyroxin), diabetes mellitus, chronic liver diseases, chronic obstructive pulmonary disease, rheumatoid arthritis, chronic kidney failure and transplantation (or immunosuppressive treatment).

Spine radiographs were performed using standardized techniques and two radiologists, blinded to all data concerning the patients, performed the semiquantitative evaluation of the radiographs using the Genant method [32] to standardize the diagnosis of fractures. Each vertebrae, including T4 to L4, were classified into one of the five grades on Genant's score. Densitometric examinations were performed with two calibrated densitometers (Dual-energy X-ray absorptiometry or DXA central) for the lumbar spine and the femoral neck. The World Health Organization definitions [33] of osteopenia and of osteoporosis were applied in both locations and the greater value was taken into account.

### Ethical Aspects

The study was approved by the Ethics Committee for Clinical Research of the Primary Care Departments of Valencia and Castellon (Regional Government of Valencia Department of Health). All of the participating women were informed of the study's characteristics and risks (basically, those associated with exposure to x-rays), and all gave signed informed consent prior to examination. Because the study data could be clinically useful, we communicated the results of the densitometric and x-ray examinations to the patients, with a recommendation to visit their primary care doctor when pertinent.

### Analysis

First, we describe the socio-demographic and clinical characteristics of the sample according to the following 4 groups: 1) absence of VFX without densitometric osteoporosis, 2) absence of VFX with densitometric osteoporosis, 3) presence of only mild VFX Genant grade 1, and 4) presence of moderate-severe VFX Genant grade 2-3. Chi-square (or Fisher exact test when pertinent) was used to assess differences among groups. Second, we perform a descriptive analysis of the PCS and MCS scores stratified by groups and characteristics of the sample. To assess the possible differences between groups Multivariable Analysis of Variance (MANOVA) was used. The relevant p-value in this analysis (variance between groups) was specified as p(groups) in the corresponding tables. Because it provides helpful information, p-values corresponding to the variance between levels of the corresponding independent variable, specified as p(*variable name*), were also included in the tables. Third, we estimate means and confidence intervals (95%CI) of the SF12 domains and the PCS and MCS scores for the 4 groups, and use the ANOVA Oneway methods to evaluate differences between groups. Totals for SF-12 domains and summary scores were weighted to represent the population age-structure of the Valencia city. Finally, we use multivariate regression analysis to analyze the independent effects of VFX and osteoporosis on the PCS scores, controlling the effect of different covariables (age, chronic conditions, obesity, hypoestrogenism antecedents, fracture antecedents and educational level). We constructed an initial model with all relevant variables and we used the backward-stepwise technique, with a removing probability of 0.10 and an entry probability of 0.05, to retain the significant factors. All analyses were performed using the STATA 10.0 (Stata Corp., College Station, Texas) statistical software.

## Results

Clinical and demographic characteristics of the participating women according to the four predefined groups of absence (with or without densitometric osteoporosis) or presence of VFX (mild or moderate-severe) in the x-ray are shown in Table 1. Relevant characteristics of the sample included 51.9% of women with densitometric osteopenia and 28.0% with densitometric osteoporosis, 72.9% with at least one chronic condition, 22.1% with antiosteoporotic treatment, and 15.6% (mild: 9.4%; moderate-severe: 6.2%) with radiological vertebral fractures (21.4% weighting the sample by the age structure of the city of Valencia). Vertebral fracture was most prevalent with older age groups, lower educational level, densitometric osteoporosis, self-referred antecedents of non-vertebral clinical fracture, and in women with antiosteoporotic treatment.

**Table 1:** Clinical and socio-demographic characteristics of the sample by osteoporosis and morphometric vertebral fracture (%)

	Without vertebral fracture		With Vertebral fracture		Total
		
	T-Score > -2.5	T-Score ≤ -2.5	Mild	Moderate-severe	
**Age group ***(p < 0.001)*					

- 50-54 years	86 (79.6)	17 (15.7)	3 (2.8)	2 (1.8)	108 (13.4)

- 55-59 years	118 (77.6)	23 (15.1)	7 (4.6)	4 (2.6)	152 (18.9)

- 60-64 years	117 (69.2)	32 (18.9)	17 (10.1)	3 (1.8)	169 (21.0)

- 65-69 years	99 (59.6)	43 (25.9)	14 (8.4)	10 (6.0)	166 (20.6)

- 70-74 years	68 (47.6)	40 (28.0)	20 (14.0)	15 (10.5)	143 (17.8)

- 75 years and older	22 (33.3)	13 (19.7)	15 (22.7)	16 (24.2)	66 (8.2)

**Educational level ***(p < 0.001)*					

- Without studies	79 (52.3)	26 (17.2)	25 (16.6)	21 (13.9)	151 (18.8)

- Primary	215 (62.5)	82 (23.8)	28 (8.1)	19 (5.5)	344 (42.8)

- Second./university	132 (69.1)	44 (23.0)	11 (5.8)	4 (2.1)	191 (23.8)

- Unknown	84 (71.2)	16 (13.6)	12 (10.2)	6 (5.1)	118 (14.6)

**Densitometry ***(p < 0.001)*					

- Normal	146 (90.1)	0 (0.0)	12 (7.4)	4 (2.5)	162 (20.1)

- Osteopenia	364 (87.3)	0 (0.0)	32 (7.7)	21 (5.0)	417 (51.9)

- Osteoporosis	0 (0.0)	168 (74.7)	32 (14.2)	25 (11.1)	225 (28.0)

**Chronic conditions **(p = 0.094)*					

- None	150 (68.8)	49 (22.5)	11 (5.0)	8 (3.7)	218 (27.1)

- 1	176 (61.8)	62 (21.7)	30 (10.5)	17 (6.0)	285 (35.5)

- 2	118 (63.4)	33 (17.7)	19 (10.2)	16 (8.6)	186 (23.1)

- 3 or more	66 (57.4)	24 (20.9)	16 (13.9)	9 (7.8)	115 (14.3)

**Antecedents of non-vertebral fracture ***(p = 0.020)*					

- No	493 (64.6)	156 (20.4)	69 (9.0)	45 (5.9)	763 (94.9)

- Yes	17 (41.5)	12 (29.3)	7 (17.1)	5 (12.2)	41 (5.1)

**Hypoestrogenism antecedents ***(p = 0.407)*					

- No	416 (64.0)	131 (20.1)	65 (10.0)	38 (5.8)	650 (80.8)

- Yes	94 (61.0)	37 (24.0)	11 (7.1)	12 (7.8)	154 (19.1)

**Obesity BMI>35 ***(p = 0.010)*					

- No	447 (62.0)	162 (22.5)	69 (9.6)	43 (6.0)	721 (89.7)

- Yes	63 (75.9)	6 (7.2)	7 (8.4)	7 (8.4)	83 (10.3)

**Antiosteoporotic treatment ***(p < 0.001)*					

- No	416 (66.4)	119 (19.0)	62 (9.9)	29 (4.6)	626 (77.9)

- Yes	94 (52.8)	49 (27.5)	14 (7.9)	21 (11.8)	178 (22.1)

**Vertebral fracture localization ***(p < 0.001)*					

- Thoracic	-	-	65 (71.4)	26 (28 .6)	91 (72.2)

- Lumbar	-	-	8 (61.5)	5 (38.5)	13 (10.3)

- Both	-	-	3 (13.6)	19(86.4)	22 (17.5)

					

TOTAL	510 (63.4)	168 (20.9)	76 (9.4)	50 (6.2)	804 (100)

All percentages by rows except in the total column (by columns). BMI: Body Mass Index. *Chronic conditions: corticoid treatment, gait abnormalities for any reason, cognitive or visual deficit, depression, gastrectomy, bowel resection, inflammatory bowel disease, thyroidectomy, diabetes mellitus, chronic liver diseases, chronic obstructive pulmonary disease, rheumatoid arthritis, chronic kidney failure and transplantation. *p-values correspond to Pearson's chi-squared test*.

PCS scores by the women's characteristics and groups are shown in Table 2. PCS scores decreased with age (from 48.5 in the 50-54 years group to 40.4 in the 75 and older group), number of chronic conditions (from 50.6 for no comorbidities to 36.9 in people with 3 or more chronic conditions), antecedents of non-vertebral fracture, hypoestrogenism antecedents, obesity, antiosteoporotic treatment, and lumbar or both thoracic and lumbar localization, and increased with educational level. PCS scores also decreased with the presence of vertebral fracture (mild: 41.6, and moderate-severe: 40.3, vs. 45.6 and 46.2 in the groups without VFX). MCS scores (Table 3) were only affected by chronic conditions (worse with more conditions) and obesity (better in women with BMI higher than 35).

**Table 2:** Physical component summary score by population characteristics

	Without vertebral fracture		With Vertebral fracture		Total
		
	T-Score > -2.5	T-Score ≤ -2.5	Mild	Mod-severe	
**Age group ***[p(model)<0.0001; p(age)<0.0001; p(groups) = 0.0405]*					

- 50-54 years	48.02	51.50	51.93	36.86	48.46

- 55-59 years	48.65	46.11	43.14	50.21	48.05

- 60-64 years	44.85	48.72	42.49	41.73	45.29

- 65-69 years	43.19	44.57	41.51	42.76	43.38

- 70-74 years	41.92	42.76	39.59	37.26	41.34

- 75 years and older	39.55	42.97	40.67	39.15	40.38

**Educational level ***[p(model)<0.0001; p(educational level)<0.0001; p(groups) = 0.0265]*					

- Without studies	40.44	42.23	39.92	36.52	40.12

- Primary	44.84	46.29	42.77	42.04	44.84

- Second./university	48.04	47.69	45.36	37.19	47.58

- Unknown	46.87	43.01	39.74	49.68	45.76

**Densitometry ***[p(model)<0.0028; p(densitometry)<0.9419; p(groups) = 0.0004]*					

- Normal	45.37	-	38.44	47.77	44.91

- Osteopenia	45.31	-	41.32	40.11	44.74

- Osteoporosis	-	45.72	43.11	39.17	44.62

Chronic conditions** *[p(model)<0.0001; p(chronic)<0.0001; p(groups) = 0.0186]*					

- None	50.61	50.78	54.00	45.49	50.63

- 1	46.34	45.43	38.41	43.01	45.11

- 2	42.30	44.90	40.96	36.65	42.14

- 3 or more	36.04	37.27	39.93	36.82	36.90

**Antecedents of non-vertebral fracture ***[p(model)<0.0058; p(non-vert. fract)<0.0001; p(groups) = 0.0010]*					

- No	45.56	46.14	41.52	40.37	45.01

- Yes	38.40	40.16	42.64	39.14	39.73

**Hypoestrogenism antecedents ***[p(model)<0.0001; p(hypoestrogenism)<0.0001; p(groups) = 0.0003]*					

- No	46.10	46.37	41.50	41.59	45.43

- Yes	41.88	43.42	42.37	36.03	41.83

**Obesity BMI>35 ***[p(model)<0.0001; p(obesity)<0.0001; p(groups) = 0.0005]*					

- No	46.34	46.17	41.68	40.77	45.52

- Yes	38.14	33.65	41.12	37.09	37.98

**Antiosteoporotic treatment ***[p(model)<0.0001; p(treatment)<0.0143; p(groups) = 0.0008]*					

- No	45.65	46.19	42.45	42.34	45.28

- Yes	43.87	44.57	37.96	37.37	42.83

**Vertebral fracture localization ***[p(model) = 0.0700; p(localization) = 0.0375; p(groups) = 0.7755]*					

- Thoracic	-	-	42.83	42.24	42.66

- Lumbar	-	-	34.61	40.19	36.76

- Both	-	-	34.12	37.56	37.09

**Total **[p(groups) = 0.0004]					

TOTAL	45.33	45.72	41.62	40.26	44.14*

* Total weighted to represent the distribution of the female population by age in the city of Valencia.

** Chronic conditions: corticoid treatment, gait abnormalities for any reason, cognitive or visual deficit, depression, gastrectomy, bowel resection, inflammatory bowel disease, thyroidectomy, diabetes mellitus, chronic liver diseases, chronic obstructive pulmonary disease, rheumatoid arthritis, chronic kidney failure and transplantation. BMI: Body Mass Index.

*p-values correspond to the multivariate analysis of variance (MANOVA)*.

**Table 3:** Mental component summary score by population characteristics

	Without vertebral fracture		With Vertebral fracture		Total
		
	T-Score > -2.5	T-Score ≤ -2.5	Mild	Mod-severe	
**Age group ***[p(model) = 0.3440; p(age) = 0.6394; p(groups) = 0.1509]*					

- 50-54 years	46.84	43.99	42.70	39.31	46.14

- 55-59 years	45.76	43.89	43.85	53.67	45.60

- 60-64 years	45.42	45.52	47.90	46.19	45.70

- 65-69 years	44.85	43.43	49.42	37.21	44.41

- 70-74 years	45.86	44.38	44.55	48.36	45.52

- 75 years and older	44.61	41.24	45.70	46.72	44.77

**Educational level ***[p(model) = 0.1164; p(educ) = 0.2030; p(groups) = 0.1340]*					

- Without studies	45.08	42.04	45.53	45.98	44.76

- Primary	45.52	43.28	45.25	44.09	44.90

- Second./university	46.01	46.07	46.62	47.45	46.09

- Unknown	45.97	45.20	49.97	47.26	46.34

**Densitometry ***[p(model) = 0.0561; p(densito) = 0.0753; p(groups) = 0.4137]*					

- Normal	45.15	-	46.97	51.09	45.43

- Osteopenia	45.86	-	47.73	46.84	46.06

- Osteoporosis	-	44.01	44.59	43.56	44.04

Chronic conditions** *[p(model)<0.0001; p(chronic)<0.0001; p(groups) = 0.0354]*					

- None	48.14	46.00	48.48	49.81	47.74

- 1	46.22	45.39	48.50	45.97	46.27

- 2	45.06	42.18	46.27	47.79	44.90

- 3 or more	39.59	38.90	40.67	36.95	39.39

**Antecedents of non-vertebral fracture ***[p(model) = 0.2081; p(antec) = 0.5708; p(groups) = 0.1379]*					

- No	45.59	44.45	45.75	46.10	45.41

- Yes	47.38	38.20	51.53	40.47	44.57

**Hypoestrogenism antecedents ***[p(model) = 0.1251; p(hypoes) = 0.2038; p(groups) = 0.1181]*					

- No	45.35	44.02	46.67	44.95	45.19

- Yes	47.01	43.95	44.04	47.91	46.09

**Obesity BMI>35 ***[p(model) = 0.0303; p(obes) = 0.0242; p(groups) = 0.2067]*					

- No	45.33	43.98	46.33	45.08	45.11

- Yes	47.95	44.82	45.94	48.39	47.59

**Antiosteoporotic treatment ***[p(model) = 0.2264; p(treatment) = 0.8042; p(groups) = 0.1425]*					

- No	45.63	44.07	46.65	45.68	45.44

- Yes	45.76	43.83	44.67	45.34	45.10

**Vertebral fracture localization ***[p(model) = 0.7076; p(loc) = 0.5582; p(groups) = 0.3955]*					

- Thoracic	-	-	46.60	44.01	45.86

- Lumbar	-	-	44.49	45.47	44.87

- Both			44.41	47.66	47.22

**Total ***[p(groups) = 0.1330]*					

TOTAL	45.66	44.01	46.29	45.54	45.29*

* Total weighted to represent the distribution of the female population by age in the city of Valencia.

** Chronic conditions: corticoid treatment, gait abnormalities for any reason, cognitive or visual deficit, depression, gastrectomy, bowel resection, inflammatory bowel disease, thyroidectomy, diabetes mellitus, chronic liver diseases, chronic obstructive pulmonary disease, rheumatoid arthritis, chronic kidney failure and transplantation. BMI: Body Mass Index.

*p values correspond to the multivariate analysis of variance (MANOVA)*.

Women's scores in the eight SF-12 domains and both summary components (total are weighted by the age structure of the Valencia female population) are shown in Table 4. Physical functioning (more than 65 in woman without fracture vs. 44 in women with moderate-severe fracture), physical role, social functioning, general health, emotional role and PCS showed statistically significant differences, usually between the moderate-severe VFX group and groups without fracture. The densitometric osteoporotic group did not show differences between groups with normal-osteopenia densitometry. The domains of bodily pain, vitality mental health and the MCP score did not show differences among groups.

**Table 4:** SF-12 domains and summary scores by presence or absence of osteoporosis and morphometric vertebral fracture

	Without vertebral fracture		With Vertebral fracture		Total*
		
	T-Score > -2.5	T-Score ≤ -2.5	Mild	Mod-severe	
Physical functioning	**65.05**	**65.62**	**57.24**	**44**	**60.46**
*p < 0.0001*	(62.14-67.96)	(60.21-71.04)	(49.22-65.25)	(34.53-53.47)	(57.45-63.48)
Physical role	**80.78**	**78.57**	**71.71**	**69.5**	**77.1**
*p = 0.0003*	(78.83-82.74)	(74.99-82.14)	(65.88-77.54)	(62.91-76.08)	(75.09-79.10)
Bodily pain	**74.61**	**74.85**	**70.06**	**70**	**74.17**
*p = 0.3741*	(70.65-79.04)	(70.65-79.04)	(63.53-81.4)	(61.63-78.36)	(71.87-76.47)
General health	**49.24**	**49.13**	**40.13**	**46**	**47.57**
*p = 0.0146*	(47.24-51.24)	(45.50-52.77)	(34.15-46.11)	(39.00-52.99)	(45.62-49.51)
Vitality	**22.15**	**22.17**	**27.3**	**19**	**21.94**
*p = 0.3147*	(19.86-24.45)	(18.36-25.97)	(20.82-33.77)	(11.88-26.11)	(19.79-24.10)
Social functioning	**86.27**	**82.29**	**77.96**	**74.5**	**81.98**
*p = 0.0003*	(81.2-87.5)	(78.50-86.07)	(71.49-84.42)	(66.97-82.02)	(79.80-84.17)
Emotional role	**86.37**	**82.66**	**83.22**	**80**	**84.19**
*p = 0.0250*	(84.78-87.95)	(79.76-85.56)	(78.36-88.08)	(73.95-86.04)	(82.47-85.91)
Mental Health	**56.91**	**54.61**	**58.55**	**58.25**	**56.87**
*p = 0.4418*	(55.11-58.70)	(51.46-57.75)	(53.90-63.19)	(52.85-63.64)	(55.08-58.66)

PCS	**45.32**	**45.72**	**41.62**	**40.25**	**44.14**
*p = 0.0004*	(44.41-46.23)	(44.01-47.43)	(39.07-44.17)	(37.25-43.25)	(43.24-45.05)
MCS	**45.65**	**44**	**46.29**	**45.54**	**45.29**
*p = 0.1330*	(44.90-46.41)	(42.69-45.32)	(44.32-48.25)	(43.00-48.08)	(44.54-46.06)

SF-12: Medical Outcomes Study Survey Form 12; PCS: Physical Component Summary; MCS: Mental Component Summary. *Total weighted to represent the distribution of the female population by age in the city of Valencia.

Results of the multivariable regression fitted to explore the independent relationship between the PCS score and VFX controlling the effect of possible confounders are shown in Table 5. From a constant of 51.83, PCS scores decrease with age groups older than 70 (-2.43 for 70-74 group and -2.97 for 75 and older), chronic conditions (-4.66, -6.79 and -11.8 according to the presence of 1, 2 or at least 3 conditions), BMI > 35 (-5.35), peripheral fracture antecedents (-3.28), and hypoestrogenism antecedents (-2.61). Controlling the effect of these variables, the presence of VFX (any grade) was independently associated with a reduction of -2.05 in the PCS score.

**Table 5:** Factors associated with Physical Component Summary score in women of 50 years and older.

		**Coef**.	95%CI	p
Age group	70-74 years	-2.43	-4.24; -0.62	0.009
		
	75 years and older	-2.97	-5.53; -0.41	0.023

Chronic conditions*	1	-4.66	-6.36; -2.95	<0.001
		
	2	-6.79	-8.73; -4.86	<0.001
		
	3 or more	-11.48	-13.74; -9.23	<0.001

Obesity (BMI>35)		-5.35	-7.57; -3.12	<0.001

Non-vertebral fracture		-3.28	-6.32; -0.24	0.034

Hypoestrogenism antecedents		-2.61	-4.30; -0.92	0.002

Vertebral fracture		-2.05	-3.97; -0.14	0.036

Constant		51.83	50.50; 53.15	<0.001

n = 804; p(F)<0.0001; r^2 ^= 0.224; Adjusted r^2 ^= 0.215. BMI: Body mass index. 95%CI: 95% Confidence Interval. *Chronic conditions: corticoid treatment, gait abnormalities for any reason, cognitive or visual deficit, depression, gastrectomy, bowel resection, inflammatory bowel disease, thyroidectomy, diabetes mellitus, chronic liver diseases, chronic obstructive pulmonary disease, rheumatoid arthritis, chronic kidney failure and transplantation.

## Discussion

In the bivariate analysis (not adjusted) mild and moderate-severe vertebral fractures were associated with a decreased HRQoL measured by the SF-12 Physical Component Summary score but not with the Mental Component Summary score, while densitometric osteoporosis with no accompanying fracture was not associated with any deterioration in HRQoL. Multivariate analysis, controlling by several confounders including age and comorbidities, retained the association between vertebral fracture and worse physical HRQoL. These results confirm that prevalent morphometric vertebral fractures are independently associated with lower scores in the physical domains of HRQoL. On the contrary, and as expected, densitometric osteoporosis without accompanying fracture was not related with HRQoL physical scores.

Regarding the literature on the topic [5,7-10,12,16,18,20-29,34], the accurate estimation of osteoporosis and VFX impact on HRQoL is difficult because the populations studied and the definitions and methods used are particularly heterogeneous: 1) Previous studies may have used population samples as in our study, but also samples from primary care patients -and, therefore with some health problems- or even samples from hospital outpatient rheumatology clinics with more severe patients; 2) fracture definitions vary from morphometric (using different techniques to identify and grade deformities) to patients' self-referred fractures or limited to VFX with clinical symptoms; 3) designs vary from cross-sectional (prevalent fractures) to prospective (incident fractures) with different temporal distances between the fracture and the HRQoL instrument administration; 4) instruments used to measure HRQoL are very different and with different clinimetric properties, and 5) while VFX are more prevalent in aged people and a substantial proportion of these individuals may have clinically relevant co-morbidities and concomitant functional limitations, study analyses do not always take into account confounders, including comorbidities or osteoporotic fractures from other localizations (i.e., hip fractures). In general, this literature suggest that the more severe the vertebral fractures (clinical, incident, referred by patients, or with samples from specialized centres with more severe patients, multiple fractures) the higher the effect on HRQoL. On the contrary, in osteoporotic patients with no fractures or only mild prevalent morphometric fractures, the effect can be minimal. Our results are consistent with this interpretation, although mild morphometric fractures (Genant grade 1) seem to affect physical domains in very similar ways to moderate-severe fractures.

PCS and MCS scores (not always age-adjusted) from studies reporting these summary components from SF-36 or SF-12 surveys [18,23-29,34] are shown in Table 6. In general, the PCS score follows the behaviour described with few differences between women with or without VFX in the case of prevalent fractures in population studies and higher in selected samples with more severe patients or incident fractures. As in our study, MCS scores, with some exceptions, were not different between women with or without VFX.

**Table 6:** Physical and Mental Component Summary scores in studies using the Medical Outcomes Study Survey Form.

Author	Country	VFX	Instrument	PCS		MCS		Comments
					
				Without VFX	With VFX	Without VFX	With VFX	
FRAVO study	Spain	Prevalent	SF12	45.3; 45.7	41.6; 40.2	45.6; 44.0	46.3; 45.5	Scores for mild and moderate-severe VFX.

Lai et al, 2010[34]	China	Prevalent	SF36	14.3	14.1; 12.7	27.8	27.7; 27.2	Scores for morphometric and clinical VFX.*

Van Schoor et al 2005[18]	Holland	Prevalent	SF12	50.0	49.5; 50.8; 42.1	55.8	55.6; 53.6; 55.0	Scores for mild, moderate and severe VFX.

Cockerill et al, 2004[27]	Europe	Prevalent Incident	SF12	43.7	41.2 (39.9)	49.1	50.8 (47.2)	Scores for incident VFX in brackets.

Hallberg et al, 2004[28]	Sweden	Incident	SF36	44.3	29.6 (34.2)	51.3	45.8 (44.3)	Scores 2 years after the incident VFX in brackets.

Falch et al, 2003[29]	Norway	Incident	SF36	46.2	31.7	46.0	46.2	Referred to hospital for clinical VFX

Adachi et al, 2001[23]	Canada	Prevalent	SF36	48	44	53	54	Morphometric subclinical VFX.

Tosteson et al, 2001[24]	USA	Prevalent	SF36	47.1	40.1	53.6	54.7	45% with clinical VFX.

Naves Diaz et al, 2001[25]	Spain	Prevalent	SF36	50	47	50	48	Population study

Hall et al, 1999[26]	Australia	Prevalent	SF36	48	36	54	50	Referred to hospital for clinical VFX

PCS: Physical Component Summary Score; MCS: Mental Component Summary Score; SF12: Medical Outcomes Survey Short-Form 12; VFX: Vertebral Fracture.

*PCS and MCS scores seem to use a non standardized range of values.

Some of the factors associated with a lower physical HRQoL are similar to those described in other studies (age, chronic conditions, and antecedents of osteoporotic fracture). Obesity has also been related to a poorer physical (not mental) HRQoL [35]. We have not identified papers adjusting for hypoestrogenism antecedents in osteoporosis or VFX quality-of-life assessment. Although climacteric symptoms may have negative effects on both the physical and mental components of the HRQoL, women with hypoestrogenism antecedents would have more marked climacteric symptoms and could also have other health problems associated with HRQoL losses.

Apart from contributing to the scarce data in Spain on HRQoL osteoporosis related, our study has other strengths. First, we use a population sample not dominated by more sick women as in studies using samples recruited in outpatient clinics or in clinical trials (typically, people at high risk of fracture). In fact, PCS and MCS scores of our weighted sample are practically identical to the SF-12 population values published for Spain [31]. Second, this is one of the larger population samples with both densitometric and spine x-ray evaluations. Third, assessment of VFX was carried out with standardized and reliable methods. Fourth, we used multivariate analysis with an extended set of covariables to control confounding.

The study also has several limitations. First, cross-sectional design does not allow the establishment of causal relationships. VFX can be a causal factor of deterioration in physical HRQoL, but limitations in physical function can also causally contribute to VFX. Second, information on chronic conditions was self-referred and although we use patient pharmacologic treatments to improve this data, figures are subject to the usual biases of data obtained from interviews. Third, our sample (broken up into four non-balanced groups and analyzed for several stratums of age, chronic conditions, etc.) has few observations in certain substratums of some groups (i.e. VFX in younger women) and some of the HRQoL estimations can be quite unstable. Therefore, HRQoL figures in the stratum-groups should be considered with caution, especially in the extreme stratums with fewer cases. Fourth, our questionnaire had no information about physical activity, a relevant variable that could have influence on osteoporosis, fractures and HRQoL. Fifth, our study used the SF12 questionnaire, a generic HRQoL measurement instrument that allow us to compare our results with many of the published studies on osteoporosis and other diseases, however it is also possible that this instrument was not responsive enough to detect small changes in HRQoL in osteoporotic patients.

After adjusting for confounding factors, our results indicate that HRQoL was significantly lower in women who have experienced prevalent osteoporotic vertebral fractures (as compared with women -osteoporotic or not- without fractures). The most clinically relevant impact on HRQoL occurred in the physical domains, with an attributable reduction of about 8%-10% in the PCS score. Although the clinical relevance of vertebral fracture has been well established for long time, these results are important for burden-of-disease and cost-of-illness studies, and also reinforce the need to reduce the underdiagnosis and undertreatment of these fractures.

## List of abbreviations

(ECOS-16): Assessment of health-related quality of life in osteoporosis; (BMI): Body mass index; (DXA): Dual-energy X-ray; (EQ5D): EuroQol 5-D; (HRQoL): Health Related Quality of Life; (MOS-SF): Medical Outcomes Study Survey Form; (MCS): Mental component summary scale; (MANOVA): Multivariable Analysis of Variance; (OPAQ): Osteoporosis Assessment Questionnaire; (OQLQ): Osteoporosis Quality of Life Questionnaire; (OPTQoL): Osteoporosis-Targeted Quality of Life Questionnaire; (PCS): Physical component summary scale; (QUALIOST): Quality of Life in Osteoporosis; (QUALEFFO): Quality of Life Questionnaire of the European Foundation for Osteoporosis; (VFX): Vertebral fractures.

## Conflict of interest

None of the sponsors played any role in the study design, the collection, analysis or interpretation of data, the writing of the manuscript, or in the decision to submit it for publication.

## Authors' contributions

JSG, SP and GSG carry out the design of the study and contributed with intellectual input in the design of this paper. BRM and GSG developed the most part of the fieldwork. IH and GSG make the analysis and written the initial drafts. All authors contributed to the writing of the manuscript, corrected draft versions and approved the final version of the manuscript.

## Supplementary Material

Additional file 1 **Dropouts in the FRAVO Study**. Dropouts by reason and age groups. Click here for file

## References

[B1] Johnell O Kanis JA An estimate of the worldwide prevalence and disability associated with osteoporotic fractures Osteoporos Int 2006 17 12 1726 33 10.1007/s00198-006-0172-4 16983459

[B2] Majumdar SR Kim N Colman I Chahal AM Raymond G Jen H Siminoski KG Hanley DA Rowe BH Incidental vertebral fractures discovered with chest radiography in the emergency department: prevalence, recognition, and osteoporosis management in a cohort of elderly patients Arch Intern Med 2005 165 8 905 9 10.1001/archinte.165.8.905 15851642

[B3] Delmas PD van de Langerijt L Watts NB Eastell R Genant H Grauer A Cahall DL IMPACT Study Group Underdiagnosis of vertebral fractures is a worldwide problem: the IMPACT study J Bone Miner Res 2005 20 4 557 63 10.1359/JBMR.041214 15765173

[B4] Guyatt GH Adachi JD Clifton J Griffith LE Epstein RS Juniper EF Quality of life issues in women with vertebral fractures due to osteoporosis Arthritis Rheum 1993 36 6 750 6 10.1002/art.1780360603 8507215

[B5] Lips P van Schoor NM Quality of life in patients with osteoporosis Osteoporos Int 2005 16 5 447 55 10.1007/s00198-004-1762-7 15609073

[B6] Lizán Tudela L Badia Llach X Quality of life evaluation for osteoporosis [In Spanish] Aten Primaria 2003 31 2 126 33 12609112 10.1016/S0212-6567(03)79150-1 PMC7684255

[B7] Peasgood T Herrmann K Kanis JA Brazier JE An updated systematic review of Health State Utility Values for osteoporosis related conditions Osteoporos Int 2009 20 6 853 68 10.1007/s00198-009-0844-y 19271098

[B8] Cook DJ Guyatt GH Adachi JD Epstein RS Juniper EF Austin PA Clifton J Rosen CJ Kessenich CR Stock JL Overdorf J Miller PD Erickson AL McCLung MR McClung BL Griffith LE Ioannidis G Development and validation of the mini-osteoporosis quality of life questionnaire (OQLQ) in osteoporotic women with back pain due to vertebral fractures. Osteoporosis Quality of Life Study Group Osteoporos Int 1999 10 3 207 13 10.1007/s001980050217 10525712

[B9] Badia X Díez-Pérez A Alvarez-Sanz C Díaz-López B Diaz-Curiel M Guillén F González-Macias J Spanish GRECO Study Group Measuring quality of life in women with vertebral fractures due to osteoporosis: a comparison of the OQLQ and QUALEFFO Qual Life Res 2001 10 4 307 17 10.1023/A:1012200508847 11763244

[B10] Lips P Cooper C Agnusdei D Caulin F Egger P Johnell O Kanis JA Kellingray S Leplege A Liberman UA McCloskey E Minne H Reeve J Reginster JY Scholz M Todd C de Vernejoul MC Wiklund I Quality of life in patients with vertebral fractures: validation of the Quality of Life Questionnaire of the European Foundation for Osteoporosis (QUALEFFO). Working Party for Quality of Life of the European Foundation for Osteoporosis Osteoporos Int 10 2 150 60 10.1007/s001980050210 10501796

[B11] Randell AG Bhalerao N Nguyen TV Sambrook PN Eisman JA Silverman SL Quality of life in osteoporosis: reliability, consistency, and validity of the Osteoporosis Assessment Questionnaire J Rheumatol 1998 25 6 1171 9 9632082

[B12] Cantarelli FB Szejnfeld VL Oliveira LM Ciconelli RM Ferraz MB Quality of life in patients with osteoporosis fractures: cultural adaptation, reliability and validity of the Osteoporosis Assessment Questionnaire Clin Exp Rheumatol 1999 17 5 547 51 10544837

[B13] Lydick E Zimmerman SI Yawn B Love B Kleerekoper M Ross P Martin A Holmes R Development and validation of a discriminative quality of life questionnaire for osteoporosis (the OPTQoL) J Bone Miner Res 1997 12 3 456 63 10.1359/jbmr.1997.12.3.456 9076589

[B14] Chandler JM Martin AR Girman C Ross PD Love-McClung B Lydick E Yawn BP Reliability of an Osteoporosis-Targeted Quality of Life Survey Instrument for use in the community: OPTQoL Osteoporos Int 1998 8 2 127 35 10.1007/BF02672508 9666935

[B15] Badia X Prieto L Roset M Díez-Pérez A Development of the ECOS-16 clinical questionnaire for the assessment of the quality of life in patients with osteoporosis [In Spanish] Med Clin (Barc) 2000 114 Suppl 3 68 75 10994567

[B16] Adachi JD Adami S Gehlbach S Anderson FA Boonen S Chapurlat RD Compston JE Cooper C Delmas P Díez-Pérez A Greenspan SL Hooven FH LaCroix AZ Lindsay R Netelenbos JC Wu O Pfeilschifter J Roux C Saag KG Sambrook PN Silverman S Siris ES Nika G Watts NB GLOW Investigators Impact of Prevalent Fractures on Quality of Life: Baseline Results From the Global Longitudinal Study of Osteoporosis in Women Mayo Clin Proc 2010 85 9 806 13 10.4065/mcp.2010.0082 20634496 PMC2931616

[B17] van Schoor NM Yu H Bobula J Lips P Cross-geographic region differences in quality of life in women with and without vertebral fracture Osteoporos Int 2009 20 10 1759 66 10.1007/s00198-009-0853-x 19238305

[B18] van Schoor NM Smit JH Twisk JW Lips P Impact of vertebral deformities, osteoarthritis, and other chronic diseases on quality of life: a population-based study Osteoporos Int 2005 16 7 749 56 10.1007/s00198-004-1744-9 15480572

[B19] Marquis P Cialdella P De la Loge C Development and validation of a specific quality of life module in post-menopausal women with osteoporosis: the QUALIOST Qual Life Res 2001 10 6 555 66 10.1023/A:1013041206433 11789555

[B20] de la Loge C Sullivan K Pinkney R Marquis P Roux C Meunier PJ Cross-cultural validation and analysis of responsiveness of the QUALIOST: QUAlity of Life questionnaire In OSTeoporosis Health Qual Life Outcomes 2005 3 69 10.1186/1477-7525-3-69 16283929 PMC1325267

[B21] Adachi JD Ioannidis G Pickard L Berger C Prior JC Joseph L Hanley DA Olszynski WP Murray TM Anastassiades T Hopman W Brown JP Kirkland S Joyce C Papaioannou A Poliquin S Tenenhouse A Papadimitropoulos EA The association between osteoporotic fractures and health-related quality of life as measured by the Health Utilities Index in the Canadian Multicentre Osteoporosis Study (CaMos) Osteoporos Int 2003 14 11 895 904 10.1007/s00198-003-1483-3 12920507

[B22] Gold DT The clinical impact of vertebral fractures: quality of life in women with osteoporosis Bone 1996 18 3 Suppl 185S 189S 10.1016/8756-3282(95)00500-5 8777086

[B23] Adachi JD Ioannidis G Berger C Joseph L Papaioannou A Pickard L Papadimitropoulos EA Hopman W Poliquin S Prior JC Hanley DA Olszynski WP Anastassiades T Brown JP Murray T Jackson SA Tenenhouse A Canadian Multicentre Osteoporosis Study (CaMos) Research Group The influence of osteoporotic fractures on health-related quality of life in community-dwelling men and women across Canada Osteoporos Int 2001 12 11 903 8 10.1007/s001980170017 11804016

[B24] Tosteson AN Gabriel SE Grove MR Moncur MM Kneeland TS Melton LJ Impact of hip and vertebral fractures on quality-adjusted life years Osteoporos Int 2001 12 12 1042 9 10.1007/s001980170015 11846331

[B25] Naves Díaz M Díaz López JB Rodríguez Rebollar A Gómez Alonso C Díaz Corte C Cannata Andía J Effect of vertebral fracture on health related quality of life in a Spanish population older than 54 years [in Spanish] Med Clin (Barc) 2001 116 14 533 5 11412619 10.1016/s0025-7753(01)71895-7

[B26] Hall SE Criddle RA Comito TL Prince RL A case-control study of quality of life and functional impairment in women with long-standing vertebral osteoporotic fracture Osteoporos Int 1999 9 6 508 15 10.1007/s001980050178 10624458

[B27] Cockerill W Lunt M Silman AJ Cooper C Lips P Bhalla AK Cannata JB Eastell R Felsenberg D Gennari C Johnell O Kanis JA Kiss C Masaryk P Naves M Poor G Raspe H Reid DM Reeve J Stepan J Todd C Woolf AD O'Neill TW Health-related quality of life and radiographic vertebral fracture Osteoporos Int 2004 15 2 113 9 10.1007/s00198-003-1547-4 14618303

[B28] Hallberg I Bachrach-Lindström M Hammerby S Toss G Ek AC Health-related quality of life after vertebral or hip fracture: a seven-year follow-up study BMC Musculoskelet Disord 2009 10 135 10.1186/1471-2474-10-135 19886998 PMC2776583

[B29] Falch JA Bentzen H Dahl AA Pain, functional level and emotional problems of women with osteoporosis and vertebral fractures [in Norwegian] Tidsskr Nor Laegeforen 2003 123 23 3355 7 14713966

[B30] Sanfélix-Genovés J Reig-Molla B Sanfélix-Gimeno G Peiró S Graells-Ferrer M Vega-Martínez M Giner V The population-based prevalence of osteoporotic vertebral fracture and densitometric osteoporosis in postmenopausal women over 50 in Valencia, Spain (the FRAVO Study) Bone 2010 47 3 610 6 20601286 10.1016/j.bone.2010.06.015

[B31] Vilagut G Valderas JM Ferrer M Garin O López-García E Alonso J Interpretation of SF-36 and SF-12 questionnaires in Spain: physical and mental components [in Spanish] Med Clin (Barc) 2008 130 19 726 35 10.1157/13121076 18570798

[B32] Genant HK Wu CY van Kuijk C Nevitt MC Vertebral fracture assessment using a semiquantitative technique J Bone Miner Res 1993 8 1137 48 10.1002/jbmr.5650080915 8237484

[B33] Naves M Díaz-López JB Gómez C Rodríguez-Rebollar A Rodríguez-García M Cannata-Andía JB The effect of vertebral fracture as a risk factor for osteoporotic fracture and mortality in a Spanish population Osteoporos Int 2003 14 520 4 10.1007/s00198-003-1405-4 12730754

[B34] Lai BM Tsang SW Lam CL Kung AW Validation of the quality of life questionnaire of the European foundation for osteoporosis (QUALEFFO-31) in Chinese Clin Rheumatol 2010 29 9 965 72 10.1007/s10067-010-1495-2 20577891 PMC2908749

[B35] Hopman WM Berger C Joseph L Barr SI Gao Y Prior JC Poliquin S Towheed T Anastassiades T CaMos Research Group The association between body mass index and health-related quality of life: data from CaMos, a stratified population study Qual Life Res 2007 16 10 1595 603 10.1007/s11136-007-9273-6 17957495

