# Supplementary material for: Impact of osteoporosis and vertebral fractures on quality-of-life. a population-based study in Valencia, Spain (The FRAVO Study)
Source: Health Qual Life Outcomes. 2011 Apr 6;9:20. doi: 10.1186/1477-7525-9-20 (PMC3080275; doi:10.1186/1477-7525-9-20)
Supplement: Additional file 1 — Dropouts in the FRAVO Study. Dropouts by reason and age groups. [file 1477-7525-9-20-S1.DOC]

**Impact of osteoporosis and vertebral fractures on quality-of-life. A population-based study in Valencia, Spain (The FRAVO Study).**

**Annexe 1. Dropouts in the FRAVO Study**

| **Figure 1. Drop-out rates and reasons** |
| --- |
| 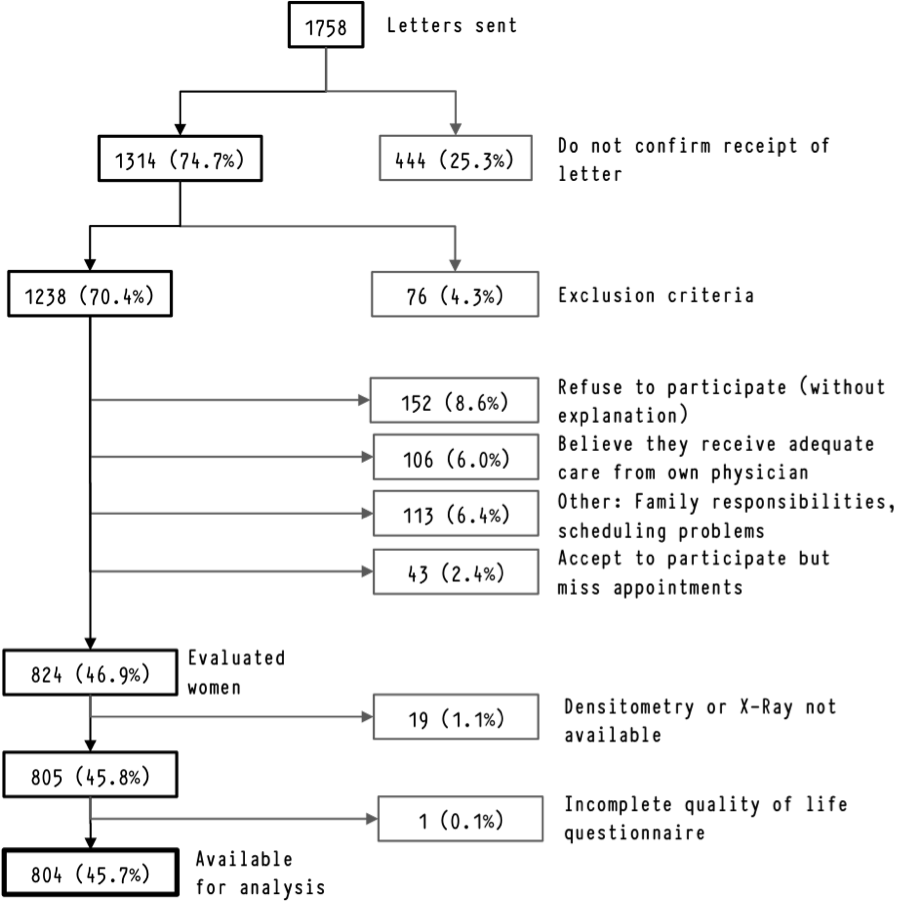 |
| Percents regarding invitation-to-participate letters sent. |

| **Drop-out rates by reason and age-groups (%)** | | | | | | | |
| --- | --- | --- | --- | --- | --- | --- | --- |
|  | **50-54** | **55-59** | **60-64** | **65-69** | **70-74** | **75+** | **Total** |
|  |  |  |  |  |  |  |  |
| Letters sent | 251 | 262 | 317 | 311 | 295 | 322 | 1758 |
|  |  |  |  |  |  |  |  |
| Did not confirm the receipt of letter | 62  (24.7) | 63  (24.0) | 80  (25.2) | 57  (18.3) | 74  (25.1) | 108  (33.5) | 444  (25.3) |
| Exclusion criteria | 36  (14.3) | 2  (0.8) | 3  (0.9) | 5  (1.6) | 3  (1.0) | 27  (8.4) | 76  (4.3) |
| Refused (without explanations) | 14  (5.6) | 13  (5.0) | 17  (5.4) | 34  (10.9) | 25  (8.5) | 49  (15.2) | 152  (8.6) |
| Believed adequate care | 10  (4.0) | 13  (5.0) | 23  (7.3) | 21  (6.7) | 22  (7.5) | 17  (5.2) | 106  (6.3) |
| Other (family, scheduling) | 9  (3.6) | 10  (3.8) | 13  (4.1) | 16  (5.1) | 22  (7.5) | 43  (13.3) | 113  (6.4) |
| missed appointments | 9  (3.6) | 5  (1.9) | 8  (2.5) | 8  (2.6) | 5  (1.7) | 8  (2.5) | 43  (2.4) |
| X-Ray or Densitometry not available | 3  (1.2) | 3  (1.1) | 4  (1.3) | 4  (1.3) | 1  (0.3) | 4  (1.2) | 19  (1.1) |
| Quality of life questionnaire incomplete | 0  (0.0) | 1  (0.4) | 0  (0.0) | 0  (0.0) | 0  (0.0) | 0  (0.0) | 1  (0.1) |
|  |  |  |  |  |  |  |  |
| Drop out (vs. letters sent) | 143  (57.0) | 110  (42.0) | 148  (46.7) | 145  (46.6) | 152  (51.5) | 256  (79.5) | 954  (54.3) |
| Drop out (vs. receipt letters) | 81  (32.3) | 47  (17.9) | 68  (21.5) | 88  (28.3) | 78  (26.4) | 148  (46.0) | 510  (29.0) |
